# Wine by-Products: Phenolic Characterization and Antioxidant Activity Evaluation of Grapes and Grape Pomaces from Six Different French Grape Varieties

**DOI:** 10.3390/molecules19010482

**Published:** 2014-01-02

**Authors:** Isabelle Ky, Bénédicte Lorrain, Natallia Kolbas, Alan Crozier, Pierre-Louis Teissedre

**Affiliations:** 1 Univ Bordeaux, ISVV, EA 4577 Œnologie, Villenave d’Ornon F-33140, France; 2 INRA, ISVV, USC 1366 Œnologie, Villenave d’Ornon F-33140, France; 3 School of Medicine, College of Medical, Veterinary & Life Sciences, University of Glasgow, Glasgow G12 8QQ, UK

**Keywords:** grape varieties, grape pomaces, grape by-products, phenolic compounds, proanthocyanidins, anthocyanins, antioxidant activity

## Abstract

Grenache, Syrah, Carignan Noir, Mourvèdre, Counoise and Alicante Bouchet grape seeds and skins, harvested in 2009 and 2010 in the Rhône valley area of France, and their respective pomaces remaining after vinification, were analyzed for their phenolic composition and antioxidant activity. The polyphenol content was quantified by HPLC and the Folin-Ciocalteu assay. The antioxidant potential was measured with four different assays: ORAC, FRAP, ABTS and DPPH. Seeds contained higher amounts of total polyphenols, up to 44.5 mg of gallic acid equivalent [GAE]/g dry weight in Alicante pomace, than skin extracts. The maximum total phenolic in skins was 31.6 mg GAE/g dry weight detected in 2010 Alicante pomace. Seeds also had the highest antioxidant capacity. HPLC analysis revealed that, despite the vinification process, pomaces still contained an appreciable amount of proanthocyanidins as well as several anthocyanin glycosides. Alicante and Syrah proved to be the varieties of most interest in terms of their potential development for nutraceutical purposes.

## 1. Introduction

Grapes of *Vitis vinifera* are one of the most cultivated fruit crops in the World, with an annual production of ~64 million metric tons in 2010 [1]. Besides the production of table grapes, grapes are used largely to produce wine and France is the second wine producer in the World after Italy [2]. Among numerous French wine appellations, after Bordeaux, the Rhône valley is the second largest in term of surface (73,468 hectares) and production (2.83 million hL). Vineyards in the Rhône valley grow Mediterranean grape cultivars, such as Grenache, which accounts for 65% of the planted area, as well as Syrah (15%), Carignan Noir (15%) and Mourvèdre (3%) [3]. Grape pomace is a rich source of polyphenols and represents an important underused residue of the wine making process. The dry grape by-product consists of pressed skins, seeds and stems and accounts for about 20% of the weight of the grapes used to make wine [4,5]. The polyphenol content of grapes and the extraction of grape polyphenols during vinification, which is far from complete, typically reaching only ca. 30%–40%, depending on grape varieties, vineyard location and technological parameters of wine making including destemming, crushing, maceration and pressing [6,7]. Grape pomace, thus, potentially constitutes a very abundant and relatively inexpensive source of a wide range of polyphenols including monomeric and oligomeric proanthocyanidins and a diversity of anthocyanin glycosides [8,9,10,11,12].

Grapes and wines which can contain high levels of phenolic antioxidants have been shown to exert beneficial effects on health [13,14,15]. For instance, polyphenolic compound in grapes are known to lower oxidative stress [16], to modulate the inflammatory cascade [17], to reduce the oxidation of LDL-c [18] and to induce protection against atherothrombotic episodes including myocardial ischemia and inhibition of platelet aggregation [19,20]. Most of these health effects have been ascribed to polyphenolic compounds serving as reducing agents in many biological systems by donating hydrogen, quenching singlet oxygen, acting as chelators and by trapping free radicals. Moreover, these antioxidant activities help to limit oxidation of nucleic acids, proteins, lipids, which may initiate degenerative diseases such as cancer, heart disease, dermal disorders and aging [21,22].

The aim of this study was to analyse polyphenolic compounds in wine by-products from six important Rhône Valley red wine cultivars: Grenache, Syrah, Carignan Noir, Mourvèdre, Counoise and Alicante Bouchet. Grape seeds and skins from these different varieties were analysed using HPLC with absorbance and fluorescence detection, and the amounts remaining in seed and skin pomaces were also evaluated. The antioxidant capacity of the grapes and pomace was assessed using four antioxidant assays (ABTS**^∙^**^+^, DPPH, FRAP and ORAC). The data may contribute to the selection of suitable seed and skin pomace for the development of antioxidant- and polyphenolic-rich nutraceuticals.

## 2. Results and Discussion

### 2.1. Grape and Pomace Composition of the Different Varieties in 2009 and 2010

#### 2.1.1. Grape and Pomace Seed Phenolic Composition in 2009 and 2010

First, TPC and total tannin content were determined (Table 1 and Table 2). For the 2009 vintage five varieties were analyzed: Grenache from two different locations (GRE1, GRE2), Syrah (SYR1), Carignan Noir (CAR), Mourvèdre (MOU) and Counoise (COU).

**Table 1:** Phenolic composition of grape seeds in 2009 and 2010.

Phenolic Composition							
**2009**							
	**GRE1 ^a^**	**GRE2 ^a^**	**SYR1 ^a^**	**CAR ^a^**	**MOU ^a^**	**COU ^a^**	
TPC	41.2 ± 1.0	42.1 ± 1.7	47.4 ± 1.0	36.6 ± 0.8	49.7 ± 4.1	42.7 ± 1.7	
Total tannins	89.8 ± 0.4	106.5 ± 4.4	89.1 ± 0.1	91.4 ± 0.8	101.0 ± 4.8	62.3 ± 1.9	
Catechin	2.33 ± 0.0	1.1 ± 0.1	1.8 ± 0.0	0.7 ± 0.0	0.43 ± 0.2	2.2 ± 0.0	
Epicatechin	1.01 ± 0.0	0.9 ± 0.1	2.3 ± 0.0	0.6 ± 0.0	0.38 ± 0.1	2.5 ± 0.1	
Σ Monomers	3.34 ± 0.0	2.1 ± 0.2	4.1 ± 0.0	1.3 ± 0.0	0.81 ± 0.3	4.7 ± 0.1	
Σ Dimers	1.03 ± 0.0	0.9 ± 0.1	0.8 ± 0.0	0.8 ± 0.0	0.49 ± 0.2	1.0 ± 0.0	
Trimer C_1_	-	-	-	-	-	-	
**Monomeric/oligomeric fraction**							
mDP	2.7 ± 0.0	2.6 ± 0.1	2.2 ± 0.0	3.1 ± 0.1	4.6 ± 0.2	2.1 ± 0.0	
%G	37.5 ± 0.0	34.9 ± 1.5	35.3 ± 1.5	36.1 ± 0.1	51.9 ± 0.7	28.7 ± 0.4	
**Polymeric fraction**							
mDP	17.6 ± 0.6	16.7 ± 0.7	13.4 ± 0.1	15.2 ± 0.7	25.1 ± 0.1	11.8 ± 0.1	
%G	58.2 ± 0.1	58.7 ± 0.3	54.4 ± 0.1	46.1 ± 0.2	58.9 ± 0.3	44.9 ± 10.8	
**2010**							
	**GRE1 ^a^**	**GRE2 ^a^**	**SYR1 ^a^**	**SYR2 ^a^**	**CAR ^a^**	**MOU ^a^**	**ALI ^a^**
TPC	88.7 ± 1.0	58.6 ± 0.2	72.8 ± 0.7	65.6 ± 0.2	58.6 ± 3.7	59.6 ± 1.5	76.4 ± 8.0
Total tannins	167.8 ± 0.9	136.8 ± 5.2	123.3 ± 1.4	115.6 ± 1.3	131.7 ± 1.4	154.9 ± 5.2	148.4 ± 7.3
Catechin	1.9 ± 0.1	3.0 ± 0.2	4.5 ± 0.1	2.9 ± 0.1	3.8 ± 0.5	2.6 ± 0.0	4.2 ± 0.0
Epicatechin	0.79 ± 0.0	1.4 ± 0.2	3.2 ± 0.2	2.9 ± 0.1	0.8 ± 0.1	0.9 ± 0.0	3.1 ± 0.3
Σ Monomers	2.6 ± 0.1	4.3 ± 0.2	7.8 ± 0.2	5.3 ± 0.1	4.6 ± 0.5	3.5 ± 0.0	7.4 ± 0.3
Σ Dimers	1.2 ± 0.1	1.7 ± 0.1	1.3 ± 0.1	1.1 ± 0.1	0.9 ± 0.1	0.6 ± 0.0	2.2 ± 0.1
Trimer C_1_	0.2 ± 0.0	0.3 ± 0.0	0.3 ± 0.0	0.3 ± 0.0	0.2 ± 0.0	0.2 ± 0.0	0.3 ± 0.0
**Monomeric/oligomeric fraction**							
mDP	2.0 ± 0.2	2.0 ± 0.2	2.02 ± 0.2	1.6 ± 0.3	3.0 ± 0.0	3.5 ± 0.5	1.7 ± 0.0
%G	48.2 ± 2.3	47.1 ± 0.1	48.7 ± 0.1	33.4 ± 5.7	55.2 ± 0.0	68.1 ± 2.5	43.3 ± 0.5
**Polymeric fraction**							
mDP	10.9 ± 0.4	11.4 ± 1.5	10.5 ± 2.0	9.9 ± 0.1	10.0 ± 0.5	13.2 ± 1.8	10.8 ± 0.5
%G	80.4 ± 8.2	92.0 ± 5.0	91.3 ± 0.2	83.3 ± 0.2	90.7 ± 4.4	92.8 ± 0.3	86.4 ± 1.6

^a^ GRE1 and GRE2, Grenache; SYR1 and SYR2, Syrah; CAR, Carignan Noir; MOU, Mourvèdre, COU, Counoise; ALI, Alicante Bouchet. In units of mg/g DW seed or skin. Data are expressed as the mean of triplicate ± standard deviation. TPC, total phenol content; Σ Monomers, sum of catechin and epicatechin; Σ Dimers, sum of dimers B_1_, B_2_, B_3_ and B_4_; mDP, mean degree of polymerization; %G, percentage of galloylation; %P, percentage of prodelphinidins; nd, not detected.

**Table 2:** Phenolic composition of grape pomace seeds in 2009 and 2010.

Phenolic Composition									
	**2009**								
	**GRE1 ^a^**		**GRE2 ^a^**	**SYR1 ^a^**	**CAR ^a^**		**MOU ^a^**		
TPC	35.3 ± 1.2		12.6 ± 1.0	30.2 ± 1.2	27.5 ± 1.0		19.0 ± 2.0		
Total tannins	105.8 ± 1.0		39.1 ± 1.2	54.3 ± 0.9	59.8 ± 0.7		51.5 ± 0.9		
Catechin	0.8 ± 0.0		0.3 ± 0.0	0.6 ± 0.1	0.3 ± 0.0		0.2 ± 0.0		
Epicatechin	0.5 ± 0.0		0.2 ± 0.0	0.8 ± 0.1	0.2 ± 0.0		0.1 ± 0.0		
Σ Monomers	1.2 ± 0.0		0.5 ± 0.0	1.4 ± 0.2	0.6 ± 0.0		0.3 ± 0.0		
Σ Dimers	0.6 ± 0.0		0.2 ± 0.0	0.4 ± 0.1	0.3 ± 0.0		0.1 ± 0.0		
Trimer C_1_	-		-	-	-		-		
**Monomeric/oligomeric fraction**									
mDP	4.0 ± 0.1		6.99 ± 0.37	2.95 ± 0.14	4.1 ± 0.0		9.2 ± 0.7		
%G	45.3 ± 0.4		48.89 ± 2.45	41.86 ± 1.96	44.2 ± 0.0		54.1± 1.0		
**Polymeric fraction**									
mDP	16.3 ± 0.6		25.8 ± 0.0	12.3 ± 0.5	19.2 ± 0.0		13.3 ± 0.0		
%G	54.9 ± 0.5		34.3 ± 0.0	53.2 ± 0.5	50.1 ± 0.8		62.8 ± 0.0		
**2010**									
	**GRE1 ^a^**	**GRE2 ^a^**	**SYR1 ^a^**	**SYR2 ^a^**	**CAR ^a^**	**MOU ^a^**		**COU ^a^**	**ALI ^a^**
TPC	40.5 ± 1.1	34.9 ± 0.2	35.6 ± 1.8	33.0 ± 1.4	38.8 ± 0.3	34.5 ± 0.1		40.8 ± 3.1	44.5 ± 0.4
Total tannins	83.1 ± 0.0	74.9 ± 1.1	79.2 ± 1.4	68.9 ± 2.3	78.7 ± 0.2	69.4 ± 3.1		70.9 ± 4.4	84.9 ± 4.0
Catechin	2.3 ± 0.0	2.3 ± 0.1	2.8 ± 0.2	1.9 ± 0.1	0.9 ± 0.1	0.4 ± 0.0		0.1 ± 0.0	4.1 ± 0.6
Epicatechin	0.0 ± 0.0	0.7 ± 0.0	2.6 ± 0.1	1.2 ± 0.0	0.4 ± 0.0	0.6 ± 0.0		2.7 ± 0.1	2.4 ± 0.4
Σ Monomers	2.4 ± 0.0	3.0 ± 0.1	5.4 ± 0.1	3.0 ± 0.1	1.3 ± 0.1	1.0 ± 0.0		2.8 ± 0.1	6.5 ± 0.9
Σ Dimers	0.7 ± 0.1	0.6 ± 0.1	0.3 ± 0.0	0.5 ± 0.1	0.5 ± 0.0	0.4 ± 0.0		0.7 ± 0.1	1.4 ± 0.1
Trimer C_1_	0.1 ± 0.0	0.1 ± 0.0	0.1 ± 0.0	0.1 ± 0.0	0.1 ± 0.0	0.1 ± 0.0		0.2 ± 0.0	0.3 ± 0.0
**Monomeric/oligomeric fraction**									
mDP	2.6 ± 0.1	2.7 ± 0.0	1.9 ± 0.2	2.3 ± 0.1	3.0 ± 0.0	4.2 ± 0.7		2.5 ± 0.3	1.9 ± 0.1
%G	62.7 ± 5.0	63.9 ± 4.1	49.6 ± 4.1	55.3± 0.3	69.7 ± 1.7	73.1 ± 3.9		57.1 ± 1.9	43.7 ± 3.0
**Polymeric fraction**									
mDP	14.6 ± 0.0	11.0 ± 1.1	8.0 ± 0.7	12.0 ± 1.8	10.4 ± 0.4	12.2 ± 1.1		11.0 ± 0.8	8.1 ± 0.1
%G	95.6 ± 0.0	92.4 ± 2.7	92.93± 2.8	92.4 ± 0.4	87.1 ± 2.0	91.7 ± 1.0		91.4 ± 2.5	88.5 ± 3.5

^a^ GRE1 and GRE2, Grenache; SYR1 and SYR2, Syrah; CAR, Carignan Noir; MOU, Mourvèdre, COU, Counoise; ALI, Alicante Bouchet. In units of mg/g DW seed or skin. Data are expressed as the mean of triplicate ± standard deviation. TPC, total phenol content; Σ Monomers, sum of catechin and epicatechin; Σ Dimers, sum of dimers B_1_, B_2_, B_3_ and B_4_; mDP, mean degree of polymerization; %G, percentage of galloylation; %P, percentage of prodelphinidins; nd, not detected.

The TPC of grape seed extracts varied slightly between varieties and ranged from 36.6 mg GAE/g DW in CAR to 49.7 mg GAE/g DW in MOU. Total tannins ranged from 62.3 mg/g DW in COU to 106.4 mg/g DW in GRE2 (Table 1). In grape pomaces (Table 2), values ranged from 12.6 mg GAE/g DW in GRE2 to 35.3 mg GAE/g DW in GRE1 for TPC and from 39.1 mg/g DW to 105.8 mg/g DW for total tannins in GRE2 and GRE1, respectively. The amount of extracted TCP was different according to grape varieties and locations (GRE1 *vs*. GRE2). Indeed, in GRE2 and MOU, up to 70% of their initial TCP was extracted during fermentation whereas in GRE1, it was only 15%.

**Figure 1:**
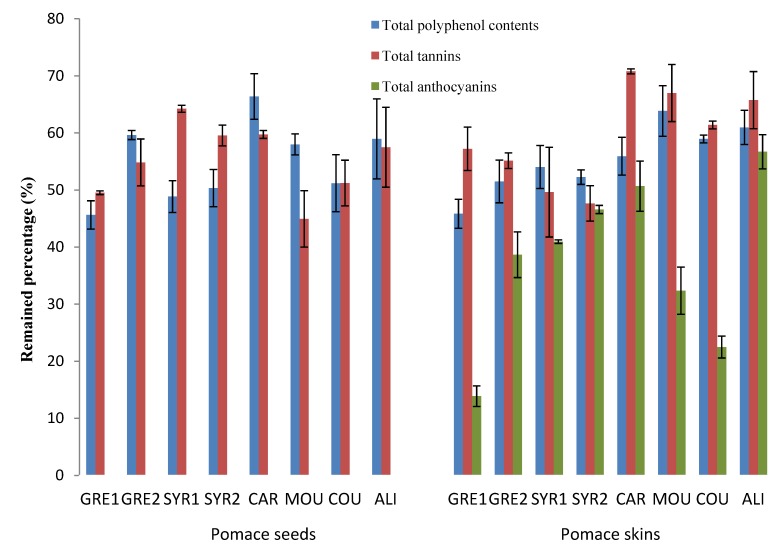
Residual phenolics (total polyphenol contents, total tannins and total anthocyanins) in 2010 grape seed and skin pomace extracts.

In the case of the 2010 vintage, two more samples were added to the study: another Syrah sample (SYR2) and Alicante (ALI). A greater variability in the amount of polyphenols can be observed in grape seeds (Table 1). The highest levels of TPC were founded in GRE1 and COU (88.7 and 83.4 mg GAE/g DW respectively) while GRE2, MOU and CAR contained lowest amounts with an average of 59 mg GAE/g DW. Total tannin levels ranged from 115.6 mg/g DW in SYR2 to 167.8 mg/g DW in GRE1. After vinification the variability was smaller ranging from 33.0 to 44.5 mg GAE/g DW for TPC and 68.9 to 84.9 mg/g DW for total tannins (Table 2). With all the varieties, more than 45% of TCPs remained in the pomace (Figure 1).

Concerning proanthocyanidin characterization, flavan-3-ol monomers [(+)-catechin, (−)-epicatechin] and oligomers (B_1_, B_2_, B_3_, B_4_ and the trimer C_1_) were identified and quantified. In grapes, for both vintages, COU contained the highest amount of monomeric and oligomeric proanthocyanidins whereas CAR and MOU had the lowest. SYR1 contained a particular rich level of monomers (4.1 mg/g DW in 2009 and 7.8 mg/g DW in 2010) while ALI was a source of an appreciable quantity of proanthocyanidins (7.4 mg/g DW of monomers, 2.2 mg/g DW of dimers and 0.34 mg/g DW of trimer C_1_) (Table 1). Regarding their respective grape pomace (Table 2), 2009 and 2010 SYR1 and ALI retained a high concentration of monomers with up to 6.5 mg/g DW remaining in ALI. The 2009 and 2010 GRE1, COU and ALI were still relatively rich in dimers. Indeed, 90% of monomers and 55% of dimers remained in GRE1 seed pomace and the respective figures for ALI, were 88% and 62%.

Concerning the 2009 monomeric/oligomeric fraction, the mDP ranged from 2.1 to 4.6 and %G from 28.7 to 51.9 while in 2010, it varied from 1.6 to 3.5 and %G from 33.3 to 68.1. Seeds from MOU were the most polymerized and galloylated followed by CAR seeds. The same trend was observed in their respective grape pomaces where MOU has a mDP of 9.2 and 4.2 and a %G of 54.1 and 73.1 in 2009 and 2010 respectively (Table 2). Compared to grapes, mDP values were higher in grape pomaces especially in 2009 with an increase of 1.3-fold of %G being observed. Indeed, as the alcohol level increases during the wine-making process, tissues become more permeable and low molecular weight tannins are released from seeds into wines toward the mid-point of fermentation and as a consequence the remaining seed pomace has a higher mDP.

For the polymeric fraction, higher values were generally observed for both vintages. MOU was still the most highly polymerized with grape mDP values ranging from 11.78 to 25.11 in 2009 and from 9.93 to 13.23 in 2010 (Table 1). These results are in keeping with those obtained in other studies with other *V*. *vinifera* varieties where mDP values of polymeric proanthocyanidins in grape seed extracts extended from 2.7 to 18.6 [23,24]. In pomaces, mDP fluctuated from 12.3 to 25.8 in 2009 and from 7.9 to 14.5 in 2010. The %G of 2010 grapes and their pomaces on average were 1.7-fold more galloylated than in 2009. No significant changes in the mDP and %G in polymeric fractions between grape and pomace from the same variety and the same vintage were observed. Values were predominantly vintage dependant.

#### 2.1.2. Grape and Pomace Skin Phenolic Composition in 2009 and 2010

The TPC, total tannin and total anthocyanin content of grape skins were analysed (Table 3 and Table 4). Samples were the same as for seed extracts and for the two vintages. As expected, grape skins contained a lower concentration of phenolic compounds than in seeds. The TPC in 2009, ranged from 20.2 mg GAE/g DW in COU to 35.5 mg GAE/g DW in SYR1 and in 2010 from 34.8 mg GAE/g DW in COU to 52.3 mg GAE/g DW in ALI (Table 3). Varieties with the highest total tannin levels were 2009 GRE1 (72.5 mg/g DW) and 2010 ALI (85.8 mg/g DW). For total anthocyanins, 2009 MOU (17.8 mg/g DW), 2009–2010 CAR (24.5 mg/g DW and 15.2 mg/g DW respectively) and 2010 ALI (18.2 mg/g DW) retained high amounts. These results are in good agreement with an earlier report on the high anthocyanin content of grapes [25].

More than 45% of TPC and total tannins remained in the grape skin pomace of all the varieties (Table 4). A different trend was observed concerning total anthocyanins, especially for MOU in 2009 and GRE1 in 2010 (Figure 1) where up to 80% of the initial amounts were extracted. Thus, anthocyanins appeared to be the most easily extractable phenolic compounds during vinification. Indeed, skins are more altered than seeds by the procedures such as pressing, crushing and maceration. During maceration, appreciable substantial quantities of anthocyanins are extracted into wine. As the level of alcohol increases during vinification, anthocyanins are solubilized and released in the acidic matrix [26].

**Table 3:** Phenolic composition of grape skins in 2009 and 2010.

Phenolic Composition						
**2009**						
	**GRE1 ^a^**	**GRE2 ^a^**	**SYR1 ^a^**	**CAR ^a^**	**MOU ^a^**	**COU ^a^**
TPC	23.4 ± 0.7	21.2 ± 0.0	35.5 ± 1.5	21.7 ± 0.4	27.3 ± 0.7	20.2 ± 0.3
Total tannins	72.5 ± 3.0	57.9 ± 1.4	66.4 ± 6.9	44.9 ± 6.1	67.2 ± 6.1	49.4 ± 0.8
Total antho	4.3 ± 0.9	10.0 ± 0.7	12.9 ± 1.4	24.5 ± 0.3	17.8 ± 2.1	13.7 ± 0.5
Catechin	0.1 ± 0.0	0.01 ± 0.0	0.3 ± 0.02	0.1 ± 0.0	0.04 ± 0.01	0.03 ± 0.0
Epicatechin	0.1 ± 0.0	0.001 ± 0.0	0.1 ± 0.06	0.02 ± 0.0	0.01 ± 0.02	0.02 ± 0.0
Σ Monomers	0.2 ± 0.0	0.01 ± 0.00	0.4 ± 0.1	0.1 ± 0.0	0.1 ± 0.01	0.1 ± 0.0
Σ Dimers	0.2 ± 0.0	0.02 ± 0.00	1.0 ± 0.1	0.1 ± 0.0	0.1 ± 0.02	0.04 ± 0.0
Trimer C_1_	-	-	-	-	-	-
**Monomeric/oligomeric fraction**						
mDP	5.7 ± 0.3	4.05 ± 0.0	1.7 ± 0.0	1.4 ± 0.0	5.3 ± 0.5	2.8 ± 0.0
%G	29.9 ± 8.5	28.3 ± 0.0	29.9 ± 0.2	18.9 ± 2.7	18.3 ± 1.1	50.7 ± 0.7
%P	47.4 ± 5.1	16.15 ± 0.0	11.3 ± 1.1	8.6 ± 0.4	52.1 ± 0.8	5.8 ± 0.2
**Polymeric fraction**						
mDP	17.3 ± 0.6	22.9 ± 0.4	18.5 ± 0.0	15.2 ± 0.7	14.1 ± 0.3	18.9 ± 0.4
%G	23.2 ± 4.0	27.4 ± 1.8	42.3 ± 2.1	46.1 ± 0.2	29.0 ± 0.2	26.6 ± 1.6
%P	16.8 ± 0.1	11.3 ± 2.2	nd	nd	nd	16.2 ± 0.0
**Anthocyanins**						
Dp	0.4 ± 0.0	nd	nd	0.4 ± 0.0	0.5 ± 0.0	nd
Cy	0.4 ± 0.0	nd	nd	0.3 ± 0.0	1.0 ± 0.0	nd
Pt	0.5 ± 0.0	0.1 ± 0.0	0.3 ± 0.00	0.4 ± 0.0	0.9 ± 0.0	0.2 ± 0.0
Pn	0.9 ± 0.0	0.3 ± 0.0	1.2 ± 0.01	0.8 ± 0.0	2.2 ± 0.0	0.5 ± 0.0
Mv	3.9 ± 0.0	0.9 ± 0.0	5.8 ± 0.05	8.1 ± 0.1	6.0 ± 0.0	0.8 ± 0.0
Σ Gly	6.1 ± 0.0	1.4 ± 0.0	7.3 ± 0.06	9.9 ± 0.1	10.6 ± 0.0	1.4 ± 0.0
Σ Ace	0.4 ± 0.0	0.2 ± 0.0	1.1 ± 0.01	0.5 ± 0.0	0.6 ± 0.0	0.2 ± 0.0
Σ Coum	0.9 ± 0.0	0.2 ± 0.0	2.7 ± 0.01	2.5 ± 0.0	1.6 ± 0.0	0.2 ± 0.0
**2010**							
	**GRE1 ^a^**	**GRE2 ^a^**	**SYR1 ^a^**	**SYR2 ^a^**	**CAR ^a^**	**MOU ^a^**	**ALI ^a^**
TPC	37.4 ± 0.7	37.9 ± 0.1	45.2 ± 2.2	39.7 ± 0.3	44.9 ± 0.1	41.3 ± 1.6	52.3 ± 3.9
Total tannins	59.5 ± 5.2	63.8 ± 0.7	73.0 ± 6.0	66.8 ± 3.3	65.2 ± 0.7	70.8 ± 3.1	85.8 ± 8.4
Total antho	11.2 ± 0.2	8.4 ± 0.7	12.1 ± 0.3	10.8 ± 0.1	15.2 ± 0.0	11.8 ± 0.2	18.2 ± 2.5
Catechin	1.2 ± 0.1	2.8 ± 0.0	1.4 ± 0.0	1.8 ± 0.0	4.0 ± 0.1	2.9 ± 0.0	7.6 ± 0.1
Epicatechin	0.3 ± 0.0	0.4 ± 0.0	0.4 ± 0.0	0.7 ± 0.1	0.3 ± 0.0	1.0 ± 0.0	1.1 ± 0.0
Σ Monomers	1.6 ± 0.0	3.2 ± 0.0	1.8 ± 0.0	2.5 ± 0.1	4.3 ± 0.0	3.9 ± 0.0	8.7 ± 0.1
Σ Dimers	0.1 ± 0.0	0.1 ± 0.0	0.0 ± 0.0	0.04 ± 0.0	0.1 ± 0.0	0.1 ± 0.0	0.3 ± 0.0
Trimer C_1_	0.004 ± 0.0	0.01 ± 0.0	0.004 ± 0.0	0.004 ± 0.0	0.002 ± 0.0	0.02 ± 0.0	0.02 ± 0.0
**Monomeric/oligomeric fraction**							
mDP	4.9 ± 0.0	5.2 ± 0.2	11.1 ± 2.0	7.4 ± 1.5	8.2 ± 0.6	6.5 ± 0.6	5.4 ± 1.2
%G	37.6 ± 6.2	30.5 ± 6.6	44.2 ± 26.1	46.6 ± 7.3	59.1 ± 2.3	47.1 ± 7.5	40.1 ± 9.6
%P	25.2 ± 6.9	19.4 ± 3.4	24.7 ± 15.7	17.7 ± 2.6	10.3 ± 1.5	14.6 ± 2.0	8.5 ± 0.7
**Polymeric fraction**							
mDP	22.4 ± 1.2	19.4 ± 0.1	21.4 ± 2.3	18.8 ± 0.0	24.9 ± 0.7	21.4 ± 0.5	22.1 ± 0.2
%G	7.7 ± 0.5	7.1 ± 0.5	6.9 ± 1.4	7.8 ± 0.0	9.0 ± 0.2	7.7 ± 0.4	8.4 ± 0. 5
%P	26.0 ± 2.8	28.5 ± 0.7	29.8 ± 3.3	26.5 ± 0.0	30.5 ± 0.4	23.5 ± 1.0	25.9 ± 1.0
**Anthocyanins**							
Dp	2.5 ± 0.3	0.7 ± 0.1	1.4 ± 0.0	1.5 ± 0.0	2.9 ± 0.1	1.2 ± 0.0	1.5 ± 0.0
Cy	0.5 ± 0.1	0.1 ± 0.0	0.1 ± 0.0	0.2 ± 0.0	0.1 ± 0.0	0.5 ± 0.0	0.1 ± 0.0
Pt	1.4 ± 0.0	0.6 ± 0.0	1.1 ± 0.0	1.0 ± 0.0	2.6 ± 0.7	1.2 ± 0.1	1.4 ± 0.0
Pn	1.6 ± 0.1	0.9 ± 0.0	1.0 ± 0.0	0.9 ± 0.0	0.7 ± 0.2	1.4 ± 0.1	2.7 ± 0.0
Mv	6.3 ± 0.5	4.1 ± 0.3	7.0 ± 0.0	4.5 ± 0.1	8.2 ± 0.3	2.5 ± 0.2	11.5 ± 0.1
Σ Gly	12.2 ± 0.0	6.4 ± 0.0	10.4 ± 0.2	8.2 ± 0.1	14.6 ± 0.0	6.8 ± 0.2	17.4 ± 0.1
Σ Ace	0.6 ± 0.0	1.0 ± 0.3	0.7 ± 0.0	0.6 ± 0.0	0.7 ± 0.0	2.8 ± 0.0	1.6 ± 0.0
Σ Coum	1.1 ± 0.0	0.7 ± 0.0	2.0 ± 0.0	1.1 ± 0.0	3.1 ± 0.1	1.6 ± 0.0	2.4 ± 0.0

^a^ GRE1 and GRE2, Grenache; SYR1 and SYR2, Syrah; CAR, Carignan Noir; MOU, Mourvèdre, COU, Counoise; ALI, Alicante Bouchet. In units of mg/g DW seed or skin. Data are expressed as the mean of triplicate ± standard deviation. TPC, total phenol content; Σ Monomers, sum of catechin and epicatechin; Σ Dimers, sum of dimers B_1_, B_2_, B_3_ and B_4_; mDP, mean degree of polymerization; %G, percentage of galloylation; %P, percentage of prodelphinidins; Total antho, total anthocyanins; Dp, delphinidin-3-*O*-monoglucoside; Cy, Cyanidin-3-*O*-monoglucoside; Pt, Petunidin-3-*O*-monoglucoside; Pn, Peonidin-3-*O*-monoglucoside; Mv, Malvidin-3-*O*-monoglucoside; Σ gly, sum of monoglucoside anthocyanins; Σ Ace, sum of petunidin-3-*O*-acetylmonoglucoside, peonidin-3-*O*-acetylmonoglucoside and malvidin-3-*O*-acetylmonoglucoside; Σ Coum, sum of peonidin-3-(6-*O*-p-coumaroyl)monoglucoside and malvidin-3-(6-*O*-p-coumaroyl)monoglucoside; nd, not detected.

Additional information was obtained when monomeric flavan-3-ols and oligomeric proanthocyanidins were analysed by HPLC. They showed substantial amounts of epicatechin, catechin, and procyanidin dimers B_2_, B_3_ and B_4_ in 2009 GRE1 and SYR1 grape skins (Table 3). In their respective skin pomaces, GRE1 and MOU retained the highest concentration of flavan-3-ols (Table 4). SYR1 and CAR were the most extracted varieties and retaining less than 10% of monomers and dimers. In 2010, grape varieties with the highest amounts of monomers and dimers in skins were the CAR, MOU and especially ALI which contained 8.7 mg/g DW of monomers and 0.3 mg/g DW of dimers. The vinification process removed more than 65% of the monomers and especially affected catechin levels (Table 4). Pomace from 2010 skins of COU and ALI were the richest in monomeric and oligomeric proanthocyanidins (Table 4 and Figure 1).

**Table 4:** Phenolic composition of grape pomace skins in 2009 and 2010.

Phenolic Composition								
**2009**								
			**GRE1 ^a^**	**GRE2 ^a^**	**SYR1 ^a^**		**CAR ^a^**	**MOU ^a^**
TPC			18.7 ± 0.1	11.8 ± 0.7	15.5 ± 0.1		22.8 ± 0.1	12.1 ± 0.8
Total tannins			53.4 ± 1.2	31.5 ± 0.4	33.0 ± 0.1		56.1 ± 0.3	31.8 ± 4.9
Total antho			3.7 ± 0.0	3.3 ± 0.5	5.1 ± 0.0		7.1 ± 0.5	3.4 ± 0.7
Catechin			0.03 ± 0.0	0.01 ± 0.0	0.01 ± 0.0		0.01 ± 0.0	0.01 ± 0.0
Epicatechin			0.003 ± 0.0	0.003 ± 0.0	0.002 ± 0.0		0.002 ± 0.0	0.01 ± 0.0
Σ Monomers			0.03 ± 0.00	0.01 ± 0.0	0.01 ± 0.0		0.01 ± 0.00	0.02 ± 0.0
Σ Dimers			0.03 ± 0.0	0.01 ± 0.0	0.01 ± 0.0		0.01 ± 0.0	0.02 ± 0.0
Trimer C_1_			-	-	-		-	-
**Monomeric/oligomeric fraction**								
mDP			3.8 ± 0.0	3.8 ± 0.1	4.0 ± 0.3		2.23 ± 0.22	7.5 ± 0.4
%G			17.2 ± 0.5	19.8 ± 1.4	19.3 ± 0.40		10.0 ± 1.5	21.3 ± 2.1
%P			6.4 ± 0.9	28.2 ± 2.3	6.6 ± 0.4		14.2 ± 6.7	17.9 ± 5.0
**Polymeric fraction**								
mDP			13.7 ± 0.4	17.6 ± 0.0	13.0 ± 0.6		12.0 ± 0.2	11.1 ± 0.0
%G			33.3 ± 0.1	20.5 ± 0.0	38.0 ± 1.0		34.8 ± 0.7	4.7 ± 0.0
%P			7.1 ± 0.8	26.9 ± 0.0	ND		ND	58.4 ± 0.0
**Anthocyanins**								
Dp			0.3 ± 0.0	0.2 ± 0.0	0.2 ± 0.0		0.7 ± 0.0	0.2 ± 0.0
Cy			0.2 ± 0.0	0.2 ± 0.0	0.2 ± 0.0		0.2 ± 0.0	0.1 ± 0.0
Pt			0.3 ± 0.0	0.3 ± 0.0	0.3 ± 0.0		0.9 ± 0.0	0.3 ± 0.0
Pn			0.4 ± 0.0	0.5 ± 0.0	0.4 ± 0.0		0.4 ± 0.0	0.3 ± 0.0
Mv			1.7 ± 0.0	2.6 ± 0.0	3.1 ± 0.1		6.8 ± 0.0	2.0 ± 0.0
Σ Gly			2.9 ± 0.0	3.8 ± 0.0	4.2 ± 0.1		9.0 ± 0.0	2.9 ± 0.0
Σ Ace			0.4 ± 0.0	0.5 ± 0.0	0.8 ± 0.0		0.4 ± 0.0	0.2 ± 0.0
Σ Coum			1.3 ± 0.0	1.4 ± 0.0	2.3 ± 0.0		5.2 ± 0.0	0.7 ± 0.0
**2010**								
	**GRE1 ^a^**	**GRE2 ^a^**	**SYR1 ^a^**	**SYR2 ^a^**	**CAR ^a^**	**MOU ^a^**	**COU ^a^**	**ALI ^a^**
TPC	17.1 ± 0.4	19.5 ± 1.0	24.3 ± 0.0	20.8 ± 0.2	25.1 ± 1.1	26.3 ± 0.3	20.5 ± 0.2	31.6 ± 1.7
Total tannins	33.9 ± 1.4	35.2 ± 0.2	35.9 ± 1.1	31.8 ± 0.1	46.2 ± 0.3	47.3 ± 1.3	37.6 ± 0.4	55.3 ± 5.7
Total antho	1.6 ± 0.1	3.24 ± 0.0	4.9 ± 0.1	5.0 ± 0.1	7.7 ± 0.5	3.8 ± 0.3	2.0 ± 0.0	10.0 ± 0.8
Catechin	0.5 ± 0.0	0.51 ± 0.1	0.4 ± 0.0	0.6 ± 0.1	0.4 ± 0.0	0.3 ± 0.0	0.7 ± 0.1	1.3 ± 0.1
Epicatechin	0.0 ± 0.0	0.26 ± 0.0	0.3 ± 0.0	0.2 ± 0.0	0.2 ± 0.0	0.2 ± 0.0	0.6 ± 0.1	1.1 ± 0.2
Σ Monomers	0.5 ± 0.0	0.8 ± 0.1	0.7 ± 0.0	0.8 ± 0.1	0.7 ± 0.0	0.5 ± 0.0	1.3 ± 0.1	2.4 ± 0.2
Σ Dimers	0.1 ± 0.0	0.1 ± 0.0	0.2 ± 0.0	0.04 ± 0.0	0.1 ± 0.0	0.1 ± 0.00	0.2 ± 0.0	0.1 ± 0.0
Trimer C_1_	0.02 ± 0.0	0.02 ± 0.0	0.03 ± 0.0	0.01 ± 0.0	0.01 ± 0.0	0.03 ± 0.0	0.04 ± 0.0	0.03 ± 0.0
**Monomeric/oligomeric fraction**								
mDP	12.1 ± 0.9	7.8 ± 0.0	9.4 ± 0.0	10.2 ± 0.0	10.3 ± 0.1	9.2 ± 0.5	11.2 ± 0.5	9.9 ± 2.4
%G	9.7 ± 0.3	19.2 ± 0.0	20.9 ± 0.8	23.8 ± 0.1	17.3 ± 0.3	18.6 ± 0.1	25.3 ± 0.3	13.8 ± 0.8
%P	16.9 ± 2.1	24.9 ± 0.0	29.1 ± 0.2	26.3 ± 0.0	34.9 ± 0.6	26.1 ± 0.3	32.4 ± 0.6	31.1 ± 0.6
**Polymeric fraction**								
mDP	10.7 ± 0.2	11.0 ± 0.8	11.5 ± 0.8	12.2 ± 1.2	12.1 ± 0.6	12.2 ± 1.2	11.7 ± 0.3	10.9 ± 1.8
%G	10.0 ± 0.5	7.6 ± 0.5	7.1 ± 0.2	10.5 ± 0.2	6.1 ± 0.0	10.5 ± 0.2	10.2 ± 0.6	11.0 ± 0.9
%P	17.7 ± 0.3	19.2 ± 0.2	19.0 ± 0.4	18.1 ± 0.1	20.7 ± 0.4	18.1 ± 0.1	18.8 ± 0.0	16.7 ± 1.0
**Anthocyanins**								
Dp	0.3 ± 0.0	1.5 ± 0.3	1.2 ± 0.0	1.5 ± 0.0	4.2 ± 0.7	1.4 ± 0.2	0.6 ± 0.0	1.6 ± 0.1
Cy	0.1 ± 0.0	0.1 ± 0.0	0.1 ± 0.0	0.1 ± 0.0	0.1 ± 0.0	0.1 ± 0.0	0.1 ± 0.0	0.1 ± 0.0
Pt	0.3 ± 0.0	0.9 ± 0.2	0.9 ± 0.0	1.0 ± 0.0	1.9 ± 0.2	0.9 ± 0.1	0.4 ± 0.0	1.2 ± 0.1
Pn	0.3 ± 0.0	0.7 ± 0.1	0.6 ± 0.0	0.6 ± 0.0	0.5 ± 0.0	0.5 ± 0.1	0.5 ± 0.0	2.6 ± 0.1
Mv	1.6 ± 0.0	5.4 ± 0.8	7.8 ± 0.1	5.3 ± 0.0	7.1 ± 0.7	3.3 ± 0.3	1.8 ± 0.0	8.9 ± 0.1
Σ Gly	2.6 ± 0.0	8.6 ± 0.0	10.5 ± 0.0	8.5 ± 0.0	13.7 ± 0.0	6.2 ± 0.0	3.3 ± 0.1	14.3 ± 0.0
Σ Ace	0.2 ± 0.0	0.2 ± 0.0	0.2 ± 0.0	0.2 ± 0.0	0.2 ± 0.0	0.2± 0.0	0.2 ± 0.0	0.20± 0.0
Σ Coum	0.4 ± 0.0	1.0 ± 0.0	5.3 ± 0.0	1.2 ± 0.10	3.1 ± 0.0	0.7 ± 0.0	0.6 ± 0.0	4.1 ± 0.0

^a^ GRE1 and GRE2, Grenache; SYR1 and SYR2, Syrah; CAR, Carignan Noir; MOU, Mourvèdre, COU, Counoise; ALI, Alicante Bouchet. In units of mg/g DW seed or skin. Data are expressed as the mean of triplicate ± standard deviation. TPC, total phenol content; Σ Monomers, sum of catechin and epicatechin; Σ Dimers, sum of dimers B_1_, B_2_, B_3_ and B_4_; mDP, mean degree of polymerization; %G, percentage of galloylation; %P, percentage of prodelphinidins; Total antho, total anthocyanins; Dp, delphinidin-3-*O*-monoglucoside; Cy, Cyanidin-3-*O*-monoglucoside; Pt, Petunidin-3-*O*-monoglucoside; Pn, Peonidin-3-*O*-monoglucoside; Mv, Malvidin-3-*O*-monoglucoside; Σ gly, sum of monoglucoside anthocyanins; Σ Ace, sum of petunidin-3-*O*-acetylmonoglucoside, peonidin-3-*O*-acetylmonoglucoside and malvidin-3-*O*-acetylmonoglucoside; Σ Coum, sum of peonidin-3-(6-*O*-p-coumaroyl)monoglucoside and malvidin-3-(6-*O*-p-coumaroyl)monoglucoside; nd, not detected.

As previously observed in previous studies [27,28] the proanthocyanidins in skins differed from those in seeds primarily by the presence of prodelphinidins, higher mDP values and lower amounts of galloylated derivatives. In the case of the 2009 vintage, substantial difference in grape skins were observed between varieties especially concerning the %G which varied from 18.3 in MOU to 50.7 in COU and the %P which ranged 5.6 in COU to 52.1 in MOU in the monomeric/oligomeric fraction. mDP values varied from 1.4 to 5.7. In the polymeric fraction, mDP ranged from 14.1 to 23, %G from 23.2 to 46.1 and only the %P of GRE1, GRE2 and COU have been detected.

In 2010 grape skins, in the monomeric/oligomeric fractions, mDP varied from 4.9 to 11.1, %G from 30.5 to 59.1 and %P from 8.5 to 25.2 (Table 3). In the polymeric fraction, mDP fluctuated from 18.8 to 24.8. For the %G and %P, non-significant differences were observed between varieties and values ranged from 6.9 to 9.5 and from 22.2 to 30.5 respectively. These results are consistent with data concerning mDP values of polymeric proanthocyanidins which can vary from 10 to ~83, depending on the fractionation technique employed, the grape variety and the vintage [29,30,31]. Compared to other studies on Italian and Bordeaux grape varieties with vintage 2008, 2009 and 2010, our %G and %P are higher. Indeed, these results can be related to the varieties and vintage effects [27,30,32]. Grape skin pomace analyses underlined that vinification affected the characteristics of proanthocyanidins in skins. Actually, for the two vintages, in grape skin pomace extracts, an increase in mDP and a decrease in %G in the monomeric/oligomeric fractions was observed. As for seeds, these values suggest that proanthocyanidins with low mDP were the most readily extracted into wines. In the polymeric fraction, the trend was opposite since mDP decreased in pomaces. This observation demonstrates that not only the small proanthocyanidins but also the more polymerized ones can be extracted from skins, probably during different periods of the vinification process, especially during the maceration period. No conclusions can be drawn concerning %P in 2009 because of varietal differences. However, in 2010, %P tended to increase in monomeric/oligomeric fractions while the opposite was observed in polymeric fractions.

The anthocyanin content of skin extracts was analysed by HPLC and the profiles obtained were in good agreement with those obtained in earlier studies with *V*. *vinifera* L. grapes [33,34]. In addition, individual anthocyanin concentrations obtained by HPLC were well correlated with estimates of total anthocyanin content. For both vintages and for all varieties, malvidin-3-*O*-monoglucoside was the major anthocyanin and accounted for 40% to 55% of total anthocyanins depending on the variety (Table 3). In 2009 grapes, we noted that SYR1, CAR and MOU contained more glycosylated, acetylated and *p*-coumaroylated anthocyanins than the other varieties. Values ranged from 1.4 mg/g DW to 10.6 mg/g DW for glycosylated anthocyanins, from 0.2 mg/g DW to 1.1 mg/g DW for acetyl-anthocyanins and from 0.2 mg/g DW to 2.7 mg/g DW for *p*-coumaroylated anthocyanins. In grape skin pomace samples, CAR and SYR1 still contained the highest amounts of glycosylated and *p*-coumaroylated anthocyanins, with 8.9 mg/g DW and 5.2 mg/g DW respectively. MOU was the most affected by vinification since more than 70% of the initial anthocyanins were extracted into wines.

As previously noted, the anthocyanin content of grape skins was higher in 2010 than 2009. Grape skin extracts from ALI contained the highest quantities of glycoside-, acetyl- and *p-*coumaroyl-anthocyanins, 17.40, 1.57 and 2.38 mg/g DW, respectively (Table 3). Indeed, “teinturier” cultivars (*i*.*e*., Alicante Bouchet) had higher anthocyanin content than “non-teinturier” grapes (*i*.*e*., Grenache, Syrah, Carignan, Mourvèdre and Carignan). It has been reported that Alicante skins contain principally malvidin-3-*O*-glucoside (39%–48% of the total) as expected for the *V*. *vinifera* cultivars, but also contain unusually high amounts of peonidin-3-*O*-glucoside (19%–31%) when compared to Cabernet Sauvignon and Tempranillo [35]. Among “non-teinturier” varieties, SYR1 and CAR were particularly rich in glycosylated and *p*-coumaroylated anthocyanins, for both 2009 and 2010 vintages, while MOU was rich in acetylated anthocyanins, especially in 2010. Appreciable amounts of anthocyanins remained in grape skin pomace of SYR1, CAR and ALI, with up to 14.3 mg/g DW, 0.22 mg/g DW and 5.3 mg/g DW of glycoside-, acetyl- and *p*-coumaroyl-anthocyanins, respectively, being retained (Table 4). In 2009 and 2010 skin pomace of GRE1 and COU contained the lowest levels of anthocyanins whereas CAR (2009 and 2010), GRE2 (2010), SYR2 (2010) and MOU (2010) retained high quantities of glycoside-, acetyl- and *p*-coumaroyl-anthocyanins.

Furthermore, for the two vintages, the data obtained with grape skins and pomace skins indicated that the wine making process resulted in a relative increase *p*-coumaroyl derivatives and a decrease of the acetyl-anthocyanins. This phenomenon has also been observed in an earlier study which found that the relative content of *p*-coumaroyl derivatives of malvidin and peonidin was lower in wines than in fresh grape skins but higher in pomace [36]. Slow rates of extraction of the *p*-coumaroyl anthocyanins compared to the acetyl-anthocyanins from skins during vinification could explained the presence of similar amounts of these anthocyanins in fresh grape skins and pomace skins [37].

Several studies have shown that phenolic composition in grapes, wines and pomaces highly depend on grape varieties, vineyard location, cultivation system, vintages and winemaking process [7,27,38,39]. Most of analytical studies have mentioned the question of “terroir” from the viticultural point of view, considering the impact of environmental factors (*i*.*e*., soil composition, climatic changes, vine phenology) on the quality of the grape or wine [39,40]. The Rhône Valley area ground consists of stony, well aerated and free-draining soil composed of a layer of marine molasses (sandstone) covered by alpine alluvium and the presence of a great number of rounded stones known as “galets” on the topsoil. These “galets” make a significant contribution to the quality of the wines by retaining the heat of the day and radiate it to the vines during the night. Considering these observations, when studying the soil alone, it is difficult to determine its influence on the constitution and the quality of grape and wine. Climatic conditions of the vintages greatly impact the grape composition [40] and factors such as the recorded climatic conditions and weather indicators (*i*.*e*., temperatures, sunlight exposure and vine water status) should be also taken into account. In the present investigation, higher concentrations of polyphenols were found in 2010 vintage seeds and skins than in 2009. Considering climatic conditions, cumulated precipitation 60 days before flowering in the Rhône Valley area was 127 mm in 2009 and 99 mm in 2010. The water deficit induced by low rain falls in 2010 could lead to an activation of the flavonoid pathway responsible for tannin and anthocyanin biosynthesis which occurs from the flowering stage and the beginning of berry growth [41]. This observation would explain the higher TPCs obtained with 2010 grapes. Other investigators have mentioned the impact of climatic conditions such as sunlight exposure and average temperatures as factors impacting polyphenol accumulation in grapes [42,43,44]. In the Rhône valley region, sunlight exposure in 2009 and 2010 were 2958 and 2753 h respectively while average temperatures from May to September were 22 °C and 22.5 °C respectively. According to Chorti *et al*. [42] sunlight exposure which is essential for grape berry ripening could be responsible for excessive sunburn, qualitative and quantitative vine damages especially on anthocyanins. The sunlight exposure in 2009 was higher than in 2010 which could also explain a lower phenolic content in 2009 grapes. This vintage impact had direct consequences on the phenolic content of grape pomaces which followed the same pattern as their parent grapes, with higher concentrations being evident in the 2010 vintage material.

### 2.2. Antioxidant Activity of Grape Pomace Extracts

The antioxidant potential of each sample was determined in order to select the most active grape pomace seeds and skins among studied varieties. Antioxidant capacity of each extracts cannot be assessed by a single method. Indeed, antioxidant measurements can be related either to the capacity of extracts to directly transfer hydrogen to a radical (DPPH or ABTS) or to act as competitors for the peroxy radicals (ORAC test) [45]. Hence, more than one type of antioxidant measurement needs to be performed to take into account the various mode of action of antioxidants [46]. In that context, the free radical scavenging capacities of seed and skin extracts were evaluated by the four tests, the FRAP, ABTS**^∙^**^+^ decolorization, DPPH and ORAC assays.

#### 2.2.1. Antioxidant Activity of Grape Pomace Seed Extracts

Concerning the 2009 vintage, only purified extracts including the monomeric/oligomeric and polymeric fractions were tested in the antioxidant assays. Different classification of pomace seed varieties was obtained. With the monomeric/oligomeric fraction, GRE1 contained the most antioxidants (ORAC: 146.3 µM TE/g DW; FRAP: 114.6 µM Fe^2+^/g DW, ABTS: 94.8 µM TE/g DW and DPPH: 56.4 TE/g DW) in all four assays (Table 5), followed by SYR1 seed extracts. These results are in accordance with tannin analysis since SYR1 and GRE1 contain appreciable amounts of TPC and tannins as well as high levels of monomeric and dimeric proanthocyanidins. 

**Table 5:** Radical scavenging capacity of grape pomace skins and seeds in 2009.

2009										
	GRE1 ^a^		GRE2 ^a^		SYR1 ^a^		CAR ^a^		MOU ^a^	
	Mean	SD	Mean	SD	Mean	SD	Mean	SD	Mean	SD
**Seeds**										
**Purified extracts**:										
**Monomeric/oligomeric fraction**										
ORAC	146.3	17.8	102.2	16.0	84.4	29.0	42.4	10.8	41.8	4.9
FRAP	114.6	11.8	53.7	2.4	106.8	17.3	74.9	7.98	60.4	3.7
ABTS	94.8	2.1	53.1	3.6	83.3	13.8	71.6	6.0	62.6	2.1
DPPH	56.4	2.6	38.0	5.7	49.7	8.7	39.8	2.2	38.7	4.4
**Polymeric fraction**										
ORAC	94.1	1.9	42.3	8.9	66.5	5.5	54.6	2.7	51.9	0.6
FRAP	185.2	1.5	59.8	6.3	118.4	2.7	138.0	15.1	93.2	1.9
ABTS	421.4	8.9	311.0	48.7	262.9	10.0	324.3	26.6	234.0	26.5
DPPH	300.9	19.4	335.6	23.0	208.4	24.1	191.0	11.8	212.7	8.8
**Skins**										
**Purified extracts**:										
**Monomeric/oligomeric fraction**										
ORAC	63.8	3.2	63.8	2.2	94.0	1.4	37.4	1.8	47.8	14.7
FRAP	57.3	6.6	13.7	2.4	53.1	3.1	91.4	2.6	15.2	0.7
ABTS	40.6	4.2	25.3	1.5	37.1	1.5	62.0	1.6	33.7	0.9
DPPH	20.5	0.6	16.6	0.6	18.8	1.5	26.9	1.4	19.3	1.6
**Polymeric fraction**										
ORAC	86.8	0.3	55.4	3.6	57.2	1.5	66.9	7.3	42.4	4.0
FRAP	120.9	6.9	35.1	4.4	78.7	0.2	112.7	14.1	78.0	13.7
ABTS	286.7	19.9	77.9	3.7	121.1	7.1	197.0	21.5	129.8	8.8
DPPH	203.1	10.6	97.0	11.0	76.1	5.9	113.8	13.4	110.7	8.1

^a^ GRE1 and GRE2, Grenache; SYR1 and SYR2, Syrah; CAR, Carignan Noir; MOU, Mourvèdre, COU, Counoise; ALI, Alicante Bouchet; SD, standard deviation. Data are expressed as the mean of triplicate ± SD; ^b^ ORAC, ABTS and DPPH are expressed as µmol Trolox/g DW and FRAP as µmol Fe^2+^/g DW.

**Figure 2:**
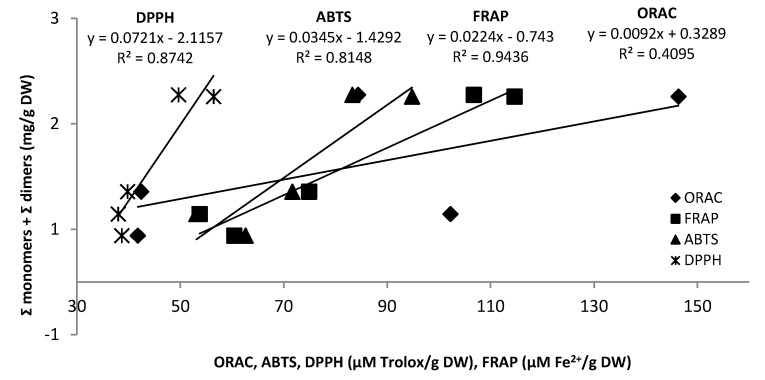
Correlations between radical scavenging capacity assays (ORAC, FRAP, ABTS and DDPH) and total proanthocyanidin content (sum of monomers and dimers) in grape pomace seeds in 2009.

Regression analyses (correlation coefficient R^2^) were attempted in order to correlate results obtained with the different methods. The best correlations were obtained with FRAP followed by DPPH and ABTS (R^2^ = 0.94, R^2^ = 0.87 and R^2^ = 0.81 respectively) (Figure 2). A weaker correlation was obtained with the ORAC assay (R^2^ = 0.41) confirming the variations in reactivity of the different assays (Figure 2). Positive correlations between TPC and antiradical activity using similar tests on grape seed samples and various plant samples have also been observed by other investigators [47,48,49,50]. In the current study, MOU was the most polymerized and galloylated sample but this feature did not seem to confer it particular higher antioxidant potential than the other samples in spite of the results of Plumb and co-workers who demonstrated that galloylated compounds have a higher antioxidant capacity in aqueous phase than their non galloylated homologues [51]. With the polymeric fraction, GRE1 again appeared as the most *in vitro* active in all four assays. No particular correlation between mDP and %G in seed pomace and antioxidant activity were evident. The antioxidant capacity in the polymeric fraction was more important than in the monomeric/oligomeric fraction, especially for the ABTS, DPPH and FRAP assays while the ORAC values showed the opposite trend.

The antioxidant assay results were similar with the 2009 and 2010 vintages (Table 6). The different tests conducted in different classifications of grapes varieties. Regarding monomeric/oligomeric fraction, ALI and COU which contained high amounts of monomeric and dimeric proanthocyanidins also had the highest antioxidant capacities in all tests. The two Syrah samples exhibited the least *in vitro* activity. Furthermore, the most polymerized and galloylated (CAR and MOU seeds) did not exert a distinctive antiradical potential. In polymeric fractions, values from FRAP, ABTS and DPPH tests were higher than those obtained with monomeric/oligomeric fractions and established ALI as the most effective variety. Results obtained from ORAC analyses varied according to varieties. As already noted, the 2009 polymeric fraction exhibited a higher antioxidant than the monomeric and oligomeric fraction, in agreement with previous reports [31,52].

#### 2.2.2. Antioxidant Activity of Grape Pomace Skin Extracts

**Figure 3:**
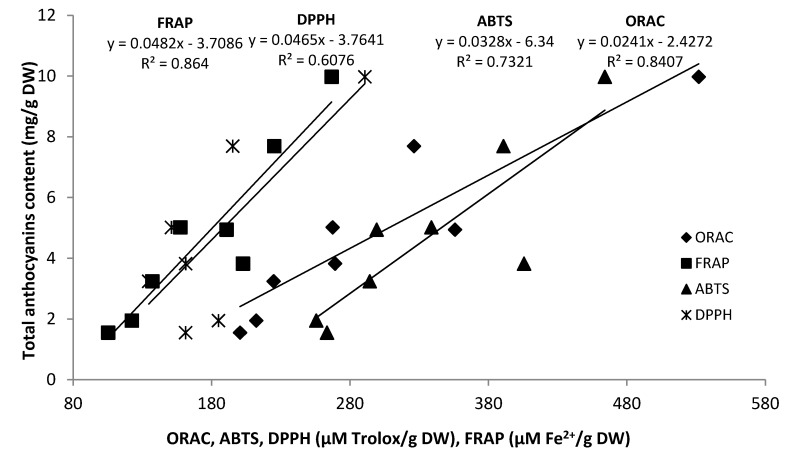
Correlations between radical scavenging capacity assays (ORAC, FRAP, ABTS and DPPH) and total anthocyanin content in grape pomace skins in 2010.

As anticipated, it was established that antioxidant activities in grape seed pomace extracts were higher than those in skins. GRE1 and CAR in 2009 (Table 5) and SYR1, CAR and ALI in 2010 contained the highest antioxidant activity (Table 6). These extracts consistently exhibited the highest values of TPC, proanthocyanidins and anthocyanins. Even though proanthocyanidin content of grape pomace skins was low, a large amount of anthocyanins still remained and this could explain their antiradical activity. The best correlations between total anthocyanins and antioxidant tests were obtained for the FRAP and ORAC tests (R^2^ = 0.86 and R^2^ = 0.84 respectively) (Figure 3) in 2010. Table 7 showed the correlation coefficients between individual anthocyanin contents and their radical scavenging capacity assays (ORAC, FRAP, ABTS and DPPH).

**Table 6:** Radical scavenging capacity of grape pomace skins and seeds in 2010.

2010																
	GRE1 ^a^		GRE2 ^a^		SYR1 ^a^		SYR2 ^a^		CAR ^a^		MOU ^a^		COU ^a^		ALI ^a^	
	Mean	SD	Mean	SD	Mean	SD	Mean	SD	Mean	SD	Mean	SD	Mean	SD	Mean	SD
**Seeds**																
**Crude extracts:**																
ORAC	322.0	20.5	303.1	45.4	266.9	10.9	267.9	18.2	248.7	14.8	201.8	16.5	327.6	34.2	561.2	29.3
FRAP	212.2	24.2	193.0	19.9	232.7	27.7	186.7	9.9	176.4	17.1	188.2	29.1	205.4	10.0	267.5	25.9
ABTS	438.1	54.2	445.7	7.2	486.3	13.0	425.8	31.6	403.5	23.0	526.4	23.8	468.6	51.9	603.1	28.5
DPPH	450.7	4.6	324.7	6.0	322.0	27.0	324.1	48.3	318.5	1.4	262.7	15.9	536.2	117.4	450.1	18.3
**Purified extracts:**																
**Monomeric/oligomeric fraction**																
ORAC	189.0	10.7	194.5	16.5	72.1	3.7	86.0	15.1	241.0	16.5	237.3	18.6	291.6	37.5	448.4	35.4
FRAP	43.8	4.7	43.11	2.1	19.0	1.9	41.3	6.8	35.6	0.8	40.2	2.7	51.6	2.7	88.9	7.0
ABTS	81.5	7.2	77.4	7.3	28.4	1.9	77.9	6.2	64.4	0.4	85.0	4.6	96.5	5.5	133.0	9.6
DPPH	39.6	4.8	41.4	2.9	13.1	1.0	35.5	3.3	31.9	1.2	42.1	2.1	48.6	0.5	73.0	4.3
**Polymeric fraction**																
ORAC	281.1	13.4	195.4	6.4	234.6	32.1	140.4	1.9	180.8	6.7	125.1	9.8	242.4	25.3	361.0	6.1
FRAP	120.1	0.7	115.8	1.5	124.5	21.1	112.0	3.0	141.1	4.8	149.2	7.5	123.3	12.5	208.3	50.9
ABTS	355.9	15.8	284.3	8.1	384.4	15.9	285.4	0.7	329.6	5.1	408.1	16.0	322.3	22.5	388.7	10.6
DPPH	268.2	7.9	236.1	10.7	246.9	42.7	212.7	11.9	229.9	3.1	301.9	5.5	282.4	28.9	338.6	10.9
**Skins**																
**Crude extracts:**																
ORAC	200.6	15.4	224.8	17.0	355.8	32.2	267.4	18.04	326.2	18.1	269.2	21.5	212.3	12.4	531.9	30.2
FRAP	105.3	4.9	137.2	2.6	190.8	6.4	157.4	11.6	225.3	5.9	202.7	9.8	122.4	8.8	266.8	40.5
ABTS	263.4	6.7	294.2	22.5	299.3	9.3	338.8	11.3	390.8	0.4	405.7	2.0	255.6	28.9	464.1	44.5
DPPH	161.2	3.7	134.6	0.5	190.6	1.5	151.0	0.6	195.3	7.4	161.3	7.1	185.0	12.6	290.7	50.1
**Purified extracts:**																
**Monomeric/oligomeric fraction**																
ORAC	82.1	3.4	83.9	4.6	154.5	11.1	87.1	9.1	73.2	7.1	72.2	8.1	133.6	11.3	204.9	24.5
FRAP	15.4	0.9	21.3	1.9	31.9	2.8	24.2	2.1	27.0	4.0	30.9	1.9	24.9	1.4	38.9	2.06
ABTS	31.7	4.4	42.0	0.1	60.1	9.1	36.9	2.7	40.2	3.0	68.2	1.4	44.3	2.0	64.6	2.4
DPPH	14.5	0.9	16.1	0.4	35.3	3.9	17.1	0.3	19.3	3.9	23.4	0.9	16.0	0.6	27.1	1.6
**Polymeric fraction**																
ORAC	137.9	6.8	132.5	5.1	142.9	15.6	117.2	1.6	148.3	3.2	125.9	6.2	99.7	2.6	266.8	26.1
FRAP	85.3	4.2	98.4	7.1	135.9	4.5	117.8	1.3	162.8	7.2	173.9	10.4	91.7	39.1	188.5	14.8
ABTS	166.1	20.4	175.2	7.6	214.1	7.5	176.7	4.7	265.5	17.6	295.9	10.9	118.0	10.1	307.6	62.0
DPPH	107.8	7.3	87.1	12.7	113.9	11.2	92.4	13.3	121.8	2.5	193.5	15.4	133.0	4.1	160.8	24.4

^a^ GRE1 and GRE2, Grenache; SYR1 and SYR2, Syrah; CAR, Carignan Noir; MOU, Mourvèdre, COU, Counoise; ALI, Alicante Bouchet; SD, standard deviation. Data are expressed as the mean of triplicate ± SD; ^b^ ORAC, ABTS and DPPH are expressed as µmol Trolox/g DW and FRAP as µmol Fe^2+^/g DW.

**Table 7:** Correlation coefficients between individual anthocyanins content and their radical scavenging capacity assays (ORAC, FRAP, ABTS and DPPH).

Correlation coefficient (R^2^) with antioxidant assays				
Individuals anthocyanins	ORAC	FRAP	ABTS	DPPH
Delphinidin-3-*O*-monoglucoside	0.12	0.34	0.27	0.26
Cyanidin-3-*O*-monoglucoside	0.19	0.3	0.42	0.23
Petunidin-3-*O*-monoglucoside	0.26	0.57	0.47	0.33
Peonidin-3-*O*-monoglucoside	0.65	0.47	0.74	0.46
Malvidin-3-*O*-monoglucoside	0.71	0.64	0.35	0.38
Petunidin-3-*O*-acetylmonoglucoside	0.53	0.26	0.74	0.62
Peonidin-3-*O*-acetylmonoglucoside ^a^	0.05	0.18	0.15	0.15
Malvidin-3-*O*-acetylmonoglucoside ^a^	0.02	0.00	0.10	0.00
Peonidin-3-(6-*O*-p-coumaroyl)monoglucoside	0.61	0.53	0.34	0.68
Malvidin-3-(6-O-p-coumaroyl)monoglucoside	0.51	0.42	0.11	0.3

^a^ Negative linear correlation values for individual anthocyanins detected by HPLC-UV.

Results showed that correlation levels were higher with total values than with the specific compound concentrations quantified by HPLC which ranged from negative values in peonidin-3-*O*-acetylmonoglucoside to R^2^ = 0.68. As noted in a recent publication [53], our result illustrate that antioxidant activity is more related to the total constituent levels than to the concentration of any individual compound despite the fact that some compounds may contribute more than the others. Moreover, our results are in accordance with those obtained by Jordáo *et al*. [50] who found negative correlation between individuals anthocyanins and ABTS antioxidant test. 

Overall, grape seed pomace exerted greater antioxidant capacities than grape skin pomace. This observation can be explained by a greater concentration in total and individual phenolic content in seeds. Furthermore, a lower amount of galloylated derivatives in skin may contributory factor as it has been demonstrated that galloylated compounds have a higher antioxidant capacity in aqueous phase than their non-galloylated homologues [51].

The discrepancy between the results obtained by different assays might be due to the conditions of the test used and/or the extract composition. Actually, as described by Huang *et al*. [46], there are numerous published methods measuring total antioxidant capacity *in vitro* and which can be classified into two types: assays based on hydrogen atom transfer (HAT) and assays based on electron transfer (ET).

HAT-based assays, like the ORAC assay, apply a competitive reaction scheme, in which antioxidant and substrate compete for thermally generated peroxyl radicals. ET-based assays measure the capacity of an antioxidant to reduce an oxidant, which changes color when reduced. The degree of color change is correlated with the sample’s antioxidant concentration. ET-based assays include the total phenols assay by DPPH and ABTS radical scavenging capacity assays and the FRAP assay. Thus, no single method is sufficient. More than one type of antioxidant capacity measurements needs to be performed to take into account various mode of action of antioxidants [46,54].

## 3. Experimental

### 3.1. Experimental Materials

#### 3.1.1. Chemicals

Deionized water was purified with a Milli-Q water system (Millipore, Bedford, MA, USA). HPLC grade acetonitrile, ethyl acetate, chloroform, methanol, ethanol and acetone purchased from Scharlau (Sentmenat, Barcelona, Spain). The following chemicals were obtained from Sigma Aldrich (St. Louis, MO, USA): (+)-catechin, (−)-epicatechin, B_1_ [(−)-epicatechin-(4β-8)-(+)-catechin], procyanidin dimer B_2_ [(−)-epicatechin-(4β-8)-(−)-epicatechin], cyanidin-3-*O*-glucoside chloride, delphinidin-3-*O*-glucoside chloride, malvidin-3-*O*-glucoside chloride, peonidin-3-*O*-glucoside chloride, gallic acid, 2,2-Diphenyl-1-picrylhydrazyl (DPPH), 6-hydroxy-2,5,7,8-tetramethylchroman-2-carboxylic acid (Trolox), 2,2'-azinobis(3-ethylbenzothiazoline-6-sulfonic acid) diammonium salt (ABTS), potassium persulfate, fluorescein, 2,2'-azobis (2-methylpropionamidine) dihydrochloride (AAPH), sodium dihydrogen phosphate dihydrate, disodium hydrogen phosphate dodecahydrate, 2,4,6-tri(2-pyridyl)-s-triazine (TPTZ), iron (III) chloride hexahydrate, iron (II) sulfate heptahydrate, Folin Ciocalteu’s phenol (2N), sodium bisulfite, sodium carbonate, phloroglucinol, L(+)-tartaric acid, L-ascorbic acid, hydrochloric acid, sodium hydroxide, acetic acid and formic acid. The Laboratory of Organic Chemistry and Organometallic (Université Bordeaux 1) synthesized procyanidins dimers B_3_ [(+)-catechin-(4α-8)-(+)-catechin], B_4_ [(+)-catechin-(4α-8)-(−)-epicatechin] and a trimer (C_1_) [(+)-catechin-(4β-8)-(+)-catechin-(4β-8)-(−)-epicatechin] [55].

#### 3.1.2. Plant Materials and Sample Preparations

This study was conducted with 2009 and 2010 grapes at maturity and their respective grape pomaces from *V*. *vinifera* L. cv. Grenache [(from two different locations (GRE1 and GRE2)], Syrah [from two different locations (SYR1 and SYR2)], Carignan (CAR), Mourvèdre (MOU), Counoise (COU) and Alicante (ALI), provided from the Rhône Valley area, appellation Châteauneuf-du-Pape. Maceration during vinification lasted during 15 and 16 days for GRE1 and GRE2 respectively, 19 and 22 days for SYR1 and SYR2 respectively, 16 days for CAR, 20 days for MOU, 11 days for COU and 11 days for ALI. GRE2, COU and ALI pomaces were analysed only for the 2010 vintage. Seeds and skins were carefully removed by hand from grapes and separated in pomace, lyophilized and stored at −20 °C prior to analysis. The dry seeds and skins were powdered in a ball grinder. Extracts of the powders were prepared in duplicate in order to obtain crude extracts according to a previous study [27,32].

#### 3.1.3. Grape and Pomace Tannins Extraction

A portion of the crude extracts (equivalent to 1 g of dried skin or seed powder from grape and pomace) was retained for further polyphenol analyses while the remaining (equivalent to 3 g of dried skin powder and 900 mg of dried seed powder) was solubilized in water/ethanol (250 mL, 95:5, *v/v*) and partitioned three times with chloroform (250 mL) to remove lipophilic material. The aqueous phase was then extracted three times with ethyl acetate (250 mL) to obtain two distinctive fractions: a low molecular weight procyanidin fraction (monomeric/oligomeric tannins) in the organic phase and a high weight procyanidin fraction (polymeric tannins) in the aqueous phase. These two fractions were concentrated and lyophilized.

#### 3.1.4. Grape and Pomace Anthocyanins Extraction

Anthocyanin extraction was adapted from the method of Sriram *et al*. [56]. A portion of dried skin powder (1 g) was extracted four times with acidified methanol (40 mL, 0.1% HCl 12N) successively for 4 h, 12 h, 4 h and 12 h. The centrifugal supernatants were combined and evaporated *in vacuo* at 30 °C to remove methanol; the residue was dissolved in water and lyophilized to obtain an anthocyanin-rich powder. 

### 3.2. Total Phenolics, Tannins and Anthocyanins

Total polyphenol, tannin and anthocyanin contents of grapes and pomace skin and seed extracts were determined. Crude extracts were solubilized in water/ethanol (90:10, *v/v*; pH 3.5 with tartaric acid) at appropriate concentrations.

TPC was determined by the Folin-Ciocalteu assay [57] and the data expressed as mg of gallic acid equivalents (GAE) per g dry weight. Total tannin content was measured by acidic hydrolysis using the method of Ribereau-Gayon and Stonestreet [58]. Anthocyanin content was determined by the SO_2_ bleaching procedure [59].

### 3.3. HPLC Analysis of Monomeric/Oligomeric Tannins

Monomeric/oligomeric tannin extracts were solubilized in a methanol/water solution (50:50, *v/v*) at appropriate concentrations and analyses were carried out according to the method of Silva *et al*. [60].

### 3.4. Determination of Mean Degree of Polymerization

The proanthocyanidin mean degree of polymerization (mDP) was determined for seed and skin extracts both in monomeric/oligomeric and polymeric tannin fractions by the means of phloroglucinolysis [61]. Analyses were carried out using the same method as described by Lorrain *et al*. [27].

### 3.5. HPLC Analysis of Anthocyanins

Powder skin extracts were dissolved in water/methanol solution (50:50, *v/v*) at a concentration of 10 mg/mL prior to UPLC-UV analyses using a Thermo-Accela HPLC system (Thermo-Fisher, San Jose, CA, USA) composed of a PDA detector, an autosampler and a quaternary 600 series pump system controlled by an Xcalibur data system. Separation was performed on a C18 Kinetex column (100 mm × 2.1 mm, 1.7 µm). The injected volume was 2 µL. The mobile phase pumped at 200 µL/min comprised a 20 min, 7%–26% gradient of acetonitrile in water with both solvents containing 5% formic acid. Eluting peaks were monitored at 520 nm. Identification of mean peaks was performed by comparison to external standards. The data was expressed as malvidin-3-*O*-monoglucoside equivalent/g dry weight of skins.

### 3.6. Antioxidant Assays

#### 3.6.1. Oxygen Radical Absorbance Capacity (ORAC) Assay

The ORAC assay was applied according to the method of Ou *et al*. [62] as modified by Dávalos *et al*. [63]. The procedure was carried out using an automated plate reader (BMG LABTECH, Ortenberg, Germany) equipped with a fluorescence detector set at excitation and emission wavelengths of 485 nm and 530 nm respectively. Analyses were conducted in a phosphate buffer (pH 7.4, 75 mM). Peroxyl radical were generated using AAPH (40 mM) and fluorescein (117 nM) was used as the substrate. Readings were taken every minute for 90 min at 37 °C. The area under the curve (AUC) was calculated for each sample by integrating the relative fluorescence curve. The net AUC was calculated by subtracting the AUC of the blank. The final ORAC values were determined by linear regression equation of Trolox concentrations and are expressed as µM Trolox equivalents/g dry weights.

#### 3.6.2. Ferric Reducing Antioxidant Potential Assay (FRAP)

FRAP assay was performed based on the method of Benzie and Strain [64] using an automated plate reader set at 593 nm. FRAP reagent were prepared daily by mixing 10 volumes of 300 mM soduim acetate buffer (pH 3.6) with 1 volume of 10 mM TPTZ solution and 1 volume 20 mM ferric chloride. A standard curve was prepared using various concentrations of FeSO_4_·7 H_2_O. Samples (40 µL) were allowed to react with FRAP reagent (300 µL) for 4 min in dark condition. Blank values were subtracted from samples and standards values then difference were used to calculate the FRAP value. Results were expressed as µM Fe^2+/^g of dry skin and seed weights.

#### 3.6.3. ABTS Assay

The ABTS assay was performed as described by Re *et al*. [65]. ABTS radical cation solution was prepared by mixing 2.45 mM of potassium persulfate and ABTS (7 mM in deionized water) following by 12–16 h incubation in the dark at room temperature. Before use, the ABTS^+•^ solution was diluted with deionized water to an absorbance of 0.7 ± 0.02 at 734 nm using a Jenway-6305 UV-vis spectrophotometer (Jenway, Staffordshire, UK). Samples (100 µL) were allowed to react with 2 mL of ABTS^+^ solution for 10 min. Blank values were subtracted from samples and standard values and a linear regression for the Trolox standards were constructed. Results were expressed as µM Trolox equivalents/g dry weights.

#### 3.6.4. DPPH Assay

This method was used according to Brand-Williams *et al*. [66] modified by Miliauskas *et al*. [67]. Samples (100 µL) were allowed to react with daily prepared DPPH**^·^** solution (2 mL, 6 × 10^−5^ M, dissolved in methanol) for 20 min at room temperature. The absorbance of the resulting solution was measured at 515 nm. Blank values were subtracted from samples and standard values. A linear regression for the Trolox standards was constructed. Results were expressed as µM Trolox equivalents/g dry weights.

### 3.7. Statistical Analysis

All measurements were performed in triplicate. Results are expressed as means ± standard deviation (SD). One-way ANOVA was performed to test the effects of variation factors (different samples) on each variable (TPC, total tannin, anthocyanin, phenol concentrations, mDP, *etc*.). If significant effects were found at a 95% confidence interval, ANOVA was followed by a Tukey’s HSD and Duncan post hoc test to identify differences among groups. These analyses were performed using Statistica V.7 Software (Statsoft Inc., Tulsa, OK, USA). Correlations among data obtained were calculated using the MS Excel software correlation coefficient statistical option.

## 4. Conclusions

This investigation screened the phenolic and antioxidant potential of by-products obtained after vinification of different Mediterranean grape varieties, in order to assess their potential for nutraceutical applications. Despite extraction during vinification, grape seed and skin pomace extracts contained appreciable amounts of flavan-3-ols and anthocyanins. The quantitative and qualitative distribution of polyphenols in grape pomaces showed significant differences through varieties and vintages. Seeds from Grenache (GRE1), Syrah (SYR1) and Alicante and skins from Syrah (SYR1), Carignan Noir and Alicante Bouchet were evidenced as the most interesting fractions because of their richest polyphenol content and highest antioxidant capacities. They, therefore represent useful by-products as a natural source of polyphenols and antioxidants for nutraceutical formulations.

## References

[1] OIV World Statistics. Proceedings of the 9th General Assembly of the OIV Porto.

[2] FAO (2010). Statistical Databases (Electronical Resource). http://faostat.fao.org.

[3] Buffin J.C. (2000). Educvin: Votre Talent de la Dégustation.

[4] Llobera A., Cañellas J. (2007). Dietary fibre content and antioxidant activity of Manto Negro red grape (*Vitis vinifera*): Pomace and stem. Food Chem..

[5] Laufenberg G., Kunz B., Nystroem M. (2003). Transformation of vegetable waste into value added products: (A) the upgrading concept; (B) practical implementations. Bioresour. Technol..

[6] Ruberto G., Renda A., Daquino C., Amico V., Spatafora C., Tringali C., Tommasi N.D. (2007). Polyphenol constituents and antioxidant activity of grape pomace extracts from five Sicilian red grape cultivars. Food Chem..

[7] Kammerer D., Claus A., Carle R., Schieber A. (2004). Polyphenol screening of pomace from red and white grape varieties (*Vitis vinifera* L.) by HPLC-DAD-MS/MS. J. Agric. Food Chem..

[8] Alonso Á.M., Guillén D.A., Barroso C.G., Puertas B., García A. (2002). Determination of antioxidant activity of wine byproducts and its correlation with polyphenolic content. J. Agric. Food Chem..

[9] Louli V., Ragoussis N., Magoulas K. (2004). Recovery of phenolic antioxidants from wine industry by-products. Bioresour. Technol..

[10] Negro C., Tommasi L., Miceli A. (2003). Phenolic compounds and antioxidant activity from red grape marc extracts. Bioresour. Technol..

[11] Xu Y., Simon J.E., Welch C., Wightman J.D., Ferruzzi M.G., Ho L., Passinetti G.M., Wu Q. (2011). Survey of polyphenol constituents in grapes and grape-derived products. J. Agric. Food Chem..

[12] Lafka T.I., Sinanoglou V., Lazos E.S. (2007). On the extraction and antioxidant activity of phenolic compounds from winery wastes. Food Chem..

[13] Teissedre P.L., Frankel E.N., Waterhouse A.L., Peleg H., German J.B. (1996). Inhibition of *in vitro* human LDL-oxidation by phenolic antioxidants from grapes and wines. J. Sci. Food Agric..

[14] Natella F., Ghiselli A., Guidi A., Ursini F., Scaccini C. (2001). Red wine mitigates the postprandial increase of LDL susceptibility to oxidation. Free Radic. Biol. Med..

[15] Gorelik S., Ligumsky M., Kohen R., Kanner J. (2008). A novel function of red wine polyphenols in humans: Prevention of absorption of cytotoxic lipid peroxidation products. FASEB J..

[16] Bagchi D., Bagchi M., Stohs S.J., Das D.K., Ray S.D., Kuszynski C.A., Joshi S.S., Pruess H.G. (2000). Free radicals and grape seed proanthocyanidin extract: Importance in human health and disease prevention. Toxicology.

[17] Castilla P., Echarri R., Dávalos A., Cerrato F., Ortega H., Teruel J.L., Lucas M.F., Gómez-Coronado D., Ortuño J., Lasunción M.A. (2006). Concentrated red grape juice exerts antioxidant, hypolipidemic, and antiinflammatory effects in both hemodialysis patients and healthy subjects. Am. J. Clin. Nutr..

[18] Sano A., Uchida R., Saito M., Shioya N., Komori Y., Tho Y., Hashizume N. (2007). Beneficial effects of grape seed extract on malondialdehyde-modified LDL. J. Nutr. Sci. Vitaminol..

[19] Arts I.C., Hollman P.C. (2005). Polyphenols and disease risk in epidemiologic studies. Am. J. Clin. Nutr..

[20] Shanmuganayagam D., Beahm M.R., Osman H.E., Krueger C.G., Reed J.D., Folts J.D. (2002). Grape seed and grape skin extracts elicit a greater antiplatelet effect when used in combination than when used individually in dogs and humans. J. Nutr..

[21] Dröge W. (2002). Free radicals in the physiological control of cell function. Physiol. Rev..

[22] Halliwell B. (1994). Free radicals, antioxidants, and human disease: Curiosity, cause, or consequence?. Lancet.

[23] Monagas M., Gómez-Cordovés C., Bartolomé B., Laureano O., da Silva R.J.M. (2003). Monomeric, oligomeric, and polymeric flavan-3-ol composition of wines and grapes from *Vitis vinifera* L. Cv. Graciano, Tempranillo, and Cabernet Sauvignon. J. Agric. Food Chem..

[24] Vidal S., Francis L., Guyot S., Marnet N., Kwiatkowski M., Gawel R., Cheynier V., Waters E.J. (2003). The mouth-feel properties of grape and apple proanthocyanidins in a wine-like medium. J. Sci. Food Agric..

[25] Jensen J.S., Demiray S., Egebo M., Meyer A.S. (2008). Prediction of wine color attributes from the phenolic profiles of red grapes (*Vitis vinifera*). J. Agric. Food Chem..

[26] Ribéreau-Gayon P., Glories Y., Maujean A., Dubourdieu D. (2006). Phenolic Compounds. Handbook of Enology: The Chemistry of Wine.Stabilization and Treatments.

[27] Lorrain B., Chira K., Teissedre P.L. (2011). Phenolic composition of Merlot and Cabernet-Sauvignon grapes from Bordeaux vineyard for the 2009-vintage: Comparison to 2006, 2007 and 2008 vintages. Food Chem..

[28] Prieur C., Rigaud J., Cheynier V., Moutounet M. (1994). Oligomeric and polymeric procyanidins from grape seeds. Phytochemistry.

[29] Souquet J.-M., Cheynier V., Brossaud F., Moutounet M. (1996). Polymeric proanthocyanidins from grape skins. Phytochemistry.

[30] Bordiga M., Travaglia F., Locatelli M., Coïsson J.D., Arlorio M. (2011). Characterisation of polymeric skin and seed proanthocyanidins during ripening in six *Vitis vinifera* L. cv.. Food Chem..

[31] Spranger I., Sun B., Mateus A.M., Freitas V.D., Ricardo-da-Silva J.M. (2008). Chemical characterization and antioxidant activities of oligomeric and polymeric procyanidin fractions from grape seeds. Food Chem..

[32] Chira K., Schmauch G., Saucier C., Fabre S., Teissedre P.L. (2009). Grape variety effect on proanthocyanidin composition and sensory perception of skin and seed tannin extracts from Bordeaux wine grapes (cabernet Sauvignon and merlot) for two consecutive vintages (2006 and 2007). J. Agric. Food Chem..

[33] Liang Z., Wu B., Fan P., Yang C., Duan W., Zheng X., Liu C., Li S. (2008). Anthocyanin composition and content in grape berry skin in *Vitis* germplasm. Food Chem..

[34] Romero-Cascales I., Ortega-Regules A., López-Roca J.M., Fernández-Fernández J.I., Gómez-Plaza E. (2005). Differences in anthocyanin extractability from grapes to wines according to variety. Am. J. Enol. Viticul..

[35] Hermosin Gutierrez I., Garcia-Romero E. (2004). Anthocyanins of red wine grape cultivars grown in the Spanish region of La Mancha: Characteristic cultivar patterns of grapes and single cultivar wines, and evolution during the ripening of the berry. Alimentaria.

[36] García-Beneytez E., Revilla E., Cabello F. (2002). Anthocyanin pattern of several red grape cultivars and wines made from them. Eur. Food Res. Technol..

[37] Fournand D., Vicens A., Sidhoum L., Souquet J.-M., Moutounet M., Cheynier V. (2006). Accumulation and extractability of grape skin tannins and anthocyanins at different advanced physiological stages. J. Agric. Food Chem..

[38] Lee J.-E., Hwang G.-S., van Den Berg F., Lee C.-H., Hong Y.-S. (2009). Evidence of vintage effects on grape wines using 1H NMR-based metabolomic study. Anal. Chim. Acta.

[39] Van Leeuwen C., Friant P., Choné X., Tregoat O., Koundouras S., Dubourdieu D. (2004). Influence of climate, soil, and cultivar on terroir. Am. J. Enol. Viticul..

[40] Pereira G.E., Gaudillere J.-P., Leeuwen C.V., Hilbert G., Maucourt M., Deborde C., Moing A., Rolin D. (2006). 1H NMR metabolite fingerprints of grape berry: Comparison of vintage and soil effects in Bordeaux grapevine growing areas. Anal. Chim. Acta.

[41] Gagné S., Lacampagne S., Claisse O., Gény L. (2009). Leucoanthocyanidin reductase and anthocyanidin reductase gene expression and activity in flowers, young berries and skins of *Vitis vinifera* L. cv. Cabernet-Sauvignon during development. Plant Physiol. Biochem..

[42] Chorti E., Guidoni S., Ferrandino A., Novello V. (2010). Effect of different cluster sunlight exposure levels on ripening and anthocyanin accumulation in Nebbiolo grapes. Am. J. Enol. Viticul..

[43] Cortell J.M., Kennedy J.A. (2006). Effect of shading on accumulation of flavonoid compounds in (*Vitis vinifera* L.) Pinot Noir fruit and extraction in a model system. J. Agric. Food Chem..

[44] Downey M.O., Harvey J.S., Robinson S.P. (2004). The effect of bunch shading on berry development and flavonoid accumulation in Shiraz grapes. Aust. J. Grape Wine Res..

[45] Roginsky V., Lissi E.A. (2005). Review of methods to determine chain-breaking antioxidant activity in food. Food Chem..

[46] Huang D., Ou B., Prior R.L. (2005). The chemistry behind antioxidant capacity assays. J. Agric. Food Chem..

[47] Bozan B., Tosun G., Özcan D. (2008). Study of polyphenol content in the seeds of red grape (*Vitis vinifera* L.) varieties cultivated in Turkey and their antiradical activity. Food Chem..

[48] Dudonné S., Vitrac X., Coutière P., Woillez M., Mérillon J.M. (2009). Comparative study of antioxidant properties and total phenolic content of 30 plant extracts of industrial interest using DPPH, ABTS, FRAP, SOD, and ORAC assays. CORD Conf. Proc..

[49] Ma X., Wu H., Liu L., Yao Q., Wang S., Zhan R., Xing S., Zhou Y. (2011). Polyphenolic compounds and antioxidant properties in mango fruits. Sci. Horticul..

[50] Jordão A.M., Correia A.C., Gonçalves F.J. (2012). Evolution of antioxidant capacity in seeds and skins during grape maturation and their association with proanthocyanidin and anthocyanin content. Vitis.

[51] Plumb G.W., de Pascual-Teresa S., Santos-Buelga C., Cheynier V., Williamson G. (1998). Antioxidant properties of catechins and proanthocyanidins: Effect of polymerisation, galloylation and glycosylation. Free Rad. Res..

[52] Es-Safi N.-E., Guyot S., Ducrot P.-H. (2006). NMR, ESI/MS, and MALDI-TOF/MS analysis of pear juice polymeric proanthocyanidins with potent free radical scavenging activity. J. Agric. Food Chem..

[53] Rockenbach I.I., Gonzaga L.V., Rizelio V.M., Gonçalves A.E.D.S.S., Genovese M.I., Fett R. (2011). Phenolic compounds and antioxidant activity of seed and skin extracts of red grape (*Vitis vinifera* and *Vitis labrusca*) pomace from Brazilian winemaking. Food Res. Int..

[54] Prior R.L., Cao G. (1999). *In vivo* total antioxidant capacity: Comparison of different analytical methods. Free Radic. Biol. Med..

[55] Tarascou I., Barathieu K., André Y., Pianet I., Dufourc E.J., Fouquet E. (2006). An improved synthesis of procyanidin dimers: Regio- and stereocontrol of the interflavan bond. Eur. J. Organ. Chem..

[56] Sriram G., Surendranath C., Sureshkumar G.K. (1999). Kinetics of anthocyanin extraction from fresh and dried grape waste. Separ. Sci. Technol..

[57] Singleton V.L., Rossi J.A. (1965). Colorimetry of total phenolics with phosphomolybdic-phosphotungstic acid reagents. Am. J. Enol. Viticul..

[58] Ribéreau Gayon P., Stonestreet E. (1966). Le dosage des tanins dans le vin rouge et détermination de leur structure. Chim. Anal..

[59] Ribéreau Gayon P., Stonestreet E. (1965). Le dosage des anthocyanes dans le vin rouge. Bull. Soc. Chim. France.

[60] Silva M.A., Ky I., Jourdes M., Teissedre P.L. (2012). Rapid and simple method for the quantification of flavan-3-ols in wine. Eur. Food Res. Technol..

[61] Drinkine J., Lopes P., Kennedy J.A., Teissedre P.L., Saucier C. (2007). Analysis of ethylidene-bridged flavan-3-ols in wine. J. Agric. Food Chem..

[62] Ou B., Hampsch-Woodill M., Prior R.L. (2001). Development and validation of an improved oxygen radical absorbance capacity assay using fluorescein as the fluorescent probe. J. Agric. Food Chem..

[63] Dávalos A., Gómez-Cordovés C., Bartolomé B. (2004). Extending applicability of the oxygen radical absorbance capacity (ORAC-Fluorescein) assay. J. Agric. Food Chem..

[64] Benzie I.F.F., Strain J.J. (1996). The ferric reducing ability of plasma (FRAP) as a measure of antioxidant power: The FRAP assay. Anal. Biochem..

[65] Re R., Pellegrini N., Proteggente A., Pannala A., Yang M., Rice-Evans C. (1999). Antioxidant activity applying an improved ABTS radical cation decolorization assay. Free Radic. Biol. Med..

[66] Brand-Williams W., Cuvelier M.E., Berset C. (1995). Use of a free radical method to evaluate antioxidant activity. LWT-Food Sci. Technol..

[67] Miliauskas G., Venskutonis P.R., van Beek T.A. (2004). Screening of radical scavenging activity of some medicinal and aromatic plant extracts. Food Chem..

